# Low-threshold topological nanolasers based on the second-order corner state

**DOI:** 10.1038/s41377-020-00352-1

**Published:** 2020-06-29

**Authors:** Weixuan Zhang, Xin Xie, Huiming Hao, Jianchen Dang, Shan Xiao, Shushu Shi, Haiqiao Ni, Zhichuan Niu, Can Wang, Kuijuan Jin, Xiangdong Zhang, Xiulai Xu

**Affiliations:** 1 Key Laboratory of advanced optoelectronic quantum architecture and measurements of Ministry of Education, School of Physics, Beijing Institute of Technology, 100081 Beijing, China; 2 Beijing Key Laboratory of Nanophotonics & Ultrafine Optoelectronic Systems, Micro-nano Center, School of Physics, Beijing Institute of Technology, 100081 Beijing, China; 3 Beijing National Laboratory for Condensed Matter Physics, Institute of Physics, Chinese Academy of Sciences, Beijing, 100190 China; 4 CAS Center for Excellence in Topological Quantum Computation and School of Physical Sciences, University of Chinese Academy of Sciences, Beijing, 100049 China; 5 State Key Laboratory of Superlattices and Microstructures, Institute of Semiconductors Chinese Academy of Sciences, Beijing, 100083 China; 6 Songshan Lake Materials Laboratory, Dongguan, Guangdong 523808 China

**Keywords:** Nanocavities, Nanophotonics and plasmonics, Quantum dots

## Abstract

Topological lasers are immune to imperfections and disorder. They have been recently demonstrated based on many kinds of robust edge states, which are mostly at the microscale. The realization of 2D on-chip topological nanolasers with a small footprint, a low threshold and high energy efficiency has yet to be explored. Here, we report the first experimental demonstration of a topological nanolaser with high performance in a 2D photonic crystal slab. A topological nanocavity is formed utilizing the Wannier-type 0D corner state. Lasing behaviour with a low threshold of approximately 1 µW and a high spontaneous emission coupling factor of 0.25 is observed with quantum dots as the active material. Such performance is much better than that of topological edge lasers and comparable to that of conventional photonic crystal nanolasers. Our experimental demonstration of a low-threshold topological nanolaser will be of great significance to the development of topological nanophotonic circuitry for the manipulation of photons in classical and quantum regimes.

## Nanophotonics: Creating better performing topological lasers

A high-performance topological laser could pave the way for its use in a wide range of nanophotonic applications. Semiconductor lasers are the most common type of laser, but their performance deteriorates if there are any structural defects in the lasing material. Topological lasers allow light to travel around a cavity of any shape without scattering, promising better performing lasers. However, creating a topological laser with a low threshold for lasing and high efficiency has proved challenging. A team of Chinese researchers led by Xiulai Xu from the Chinese Academy of Sciences have now developed a topological laser made from a two-dimensional photonic crystal nanocavity slab with a lasing threshold of about one micro-watt and high spontaneous emission coupling factor of 0.25 and is comparable to the performance of conventional semiconductor lasers.

## Introduction

The investigation of topological photonics has become one of the most fascinating frontiers in recent years^1–12^. In addition to conventional passive and linear systems, exploring topological phenomena in highly nonlinear environments also has significance^13–23^. Recently, the concept of topological lasers has been proposed and demonstrated^13–15^. These lasers exhibit topologically protected transport with the help of the robust 1D edge state in 2D systems. The pioneering work focused on the study of lasing in nonreciprocal topological cavities formed by a closed quantum-Hall-like edge state at telecommunication wavelengths^13^. However, due to the weak magneto-optic effect, the topological bandgap is only approximately 40 pm. The first magnet-free scheme for the realization of single-mode topological lasers was based on an array of ring resonators in 2D, where notably higher slope efficiencies are observed compared to the trivial counterparts^14,15^. In addition to the 1D edge state, the 0D boundary states existing in 1D lattices with nontrivial topological phases^16–20^ and the topological bulk state around the band edge^21^ have also been used to realize topological lasers. The currently designed topological lasing systems are almost at the microscale, leading to large thresholds, which are usually approximately several milliwatts. In contrast, topological nanolasers can combine the advantages of topological robustness and nanolasers, including a small footprint, a low threshold, and a high energy efficiency^24–31^, but are still lacking except for the scheme using the 0D interface state in the 1D photonic beam with a threshold of approximately 46 μW^19^.

Recently, a new class of symmetry-protected higher-order topological insulators has been proposed^32–44^. These insulators have lower-dimensional boundary states and obey a generalization of the standard bulk-boundary correspondence. In 2D cases, the 0D second-order corner state can usually be formed by two mechanisms. One is related to quantized bulk quadrupole polarization^32–37^, and the other is derived from the edge dipole polarization quantized by the 2D Zak phase^39–41^. The latter model can be easily implemented in the compact magnet-free optical platform^40^ and used to construct topological nanocavities^41^. The problem is whether we can exploit the topological nanocavity with a high quality (Q) factor and a small mode volume comparable to those of the conventional photonic crystal (PhC) nanocavity^45^ to realize a topological nanolaser with a low threshold and a high energy efficiency.

In this work, we report the first experimental demonstration of a topological nanolaser in a 2D topological PhC nanocavity, which sustains the Wannier-type 0D corner state at the nanoscale. The corner state is induced by the edge dipole polarization quantized by the 2D Zak phase. By suitably tuning the gap distance between the trivial and nontrivial parts of the PhC slab, a higher Q factor can be achieved. The robustness of the corner state with respect to defects in the bulk of the PhC is demonstrated. Lasing behaviour of the corner state with a high performance, including a low threshold and a high spontaneous emission coupling factor (*β*), is observed at 4.2 K with InGaAs quantum dots (QDs) serving as the active material. The high performance of the topological nanolaser is comparable to that of conventional semiconductor nanolasers^24–27^, indicating the great prospects of topological nanocavities for a wide range of applications in topological nanophotonic circuitry.

## Results

**Fig. 1:**
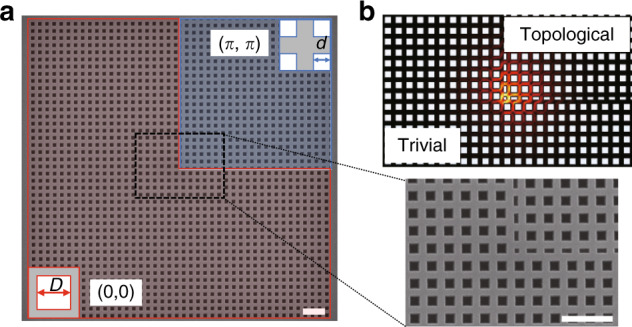
Design of the topological nanocavity. **a** Scanning electron microscopy image of a fabricated 2D topological PhC cavity in a square shape. The inset on the right shows an enlarged image around the corner. The scale bar is 1µm. The topological nanocavity consists of two topologically distinct PhCs, which are indicated by the red and blue areas. They have different unit cells, as shown in the insets. *d* and *D* are the lengths of the squares in the blue and red unit cells, in which *D*=2*d*. **b** Electric field profile of the topological corner state

Inspired by the generalized 2D SSH model, a topological nanocavity is designed, as shown in Fig. 1a. It consists of two kinds of PhC slab with square air holes, which have the same period *a* but different unit cells, as indicated by the red and blue areas in Fig. 1a. These two regions share a common band structure but possess different topologies, which are characterized by the 2D Zak phase *θ*^*Zak*^, a quantity defined by the integration of the Berry connection within the first Brillouin zone^46,47^. The PhC in the blue (red) area has a nontrivial (trivial) 2D Zak phase of *θ*^Zak^ = (*π*,*π*) (*θ*^Zak^ = (0,0)). According to the bulk-edge-corner correspondence, the mid-gap 0D corner state can be induced at the intersection of two boundaries with nonzero edge polarizations. It is worth noting that the PhC has long-range and complex couplings, different from the ideal 2D SSH mode with only nearest-neighbour couplings. Therefore, in PhC structures, the chiral symmetry is broken, and the corner state moves out of the bulk band and falls inside the bandgap. More discussions about the band structure and spatial distribution of the corner, edge, and bulk modes are shown in the Supplementary Information in detail. Figure 1b shows the electric field profile of the corner state, which is highly confined at the nanoscale. It can greatly enhance the light–matter interaction, thus having potential applications such as the construction of topological nanolasers.

**Fig. 2:**
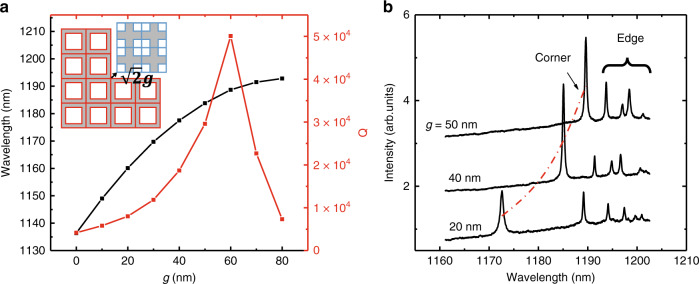
Q factors and wavelengths of the corner state for different values of *g*. **a** Calculated Q factors (red) and wavelengths (black) of the corner state for different *g*. Other parameters for these cavities are *a*=380nm and *D*=242nm. The inset shows a schematic of Q optimization, in which the topological PhC is shifted away from the corner by $\sqrt 2 g$ along the diagonal direction. **b** PL spectra for cavities with *a*=380nm, *D*=242nm and different *g*. The red dashed line represents the corner state. These peaks in the long-wavelength range originate from edge states. The PL spectra are shifted for clarity

To improve the laser performance, the Q factor of the corner state is optimized by suitably tuning the gap distance (*g*) between the trivial and nontrivial parts of the PhC slab, as shown in the inset of Fig. 2a. The black and red lines in Fig. 2a represent the calculated results of the Q factor and resonance wavelength of the corner state for different values of *g*. It is clearly shown that the Q factor first increases and then decreases as *g* gradually increases; meanwhile, the corner state shows a redshift. When *g* is initially increased, the spatial distribution of the corner state becomes smoother, decreasing the radiation loss induced by the expanded plane wave above the light cone. In this case, the Q factor increases^45^. When *g* is further increased, the transverse loss caused by the finite size effect becomes larger, which leads to a decrease in Q. Balanced by these two factors, when *g* = 60 nm, the corner mode supports a high Q factor of approximately 50,000 and a small mode volume of 0.61(*λ*/*n*)^3^, which are close to those of traditional nanocavities^45,48,49^. It is worth noting that the Q factor and mode volume of the corner state can both be disturbed by introducing perturbations around the corner. Nevertheless, the corner state always survives even with harsh perturbations to the bulk of the PhC in that the topological properties of the bulk band are not changed, which could be a practical advantage for robust applications.

We fabricated the designed topological nanocavity with different parameters in a 160-nm-thick GaAs slab (see Materials and Methods). A scanning electron microscopy image of a fabricated cavity is shown in Fig. 1a, and the inset on the right shows an enlarged image around the corner. Figure 2b shows photoluminescence (PL) spectra for cavities with different values of *g*, in which the corner states and edge states are indicated. With increasing *g* from 20 to 50 nm, the corner state exhibits a redshift, and the corresponding Q factor increases, consistent with the numerical results in Fig. 2a. However, limited by unavoidable fabrication imperfections, the fabricated Q factors are approximately an order of magnitude lower than the theoretical prediction, exhibiting usual values of 2500–5000 for cavities with *g* = 50 nm.

**Fig. 3:**
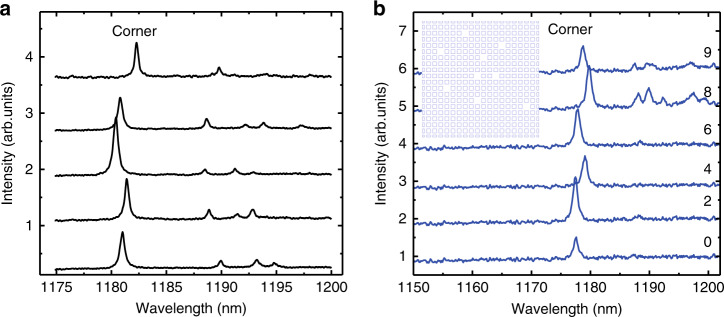
PL spectra for cavities without and with defects. **a** PL spectra of defect-free cavities with the parameters of *a*=380nm, *D*=242nm and *g*=50nm. **b** PL spectra of cavities with different numbers of defects, as shown in the inset. The numbers represent the number of missing square holes in the bulk of the PhC. Here, the missing square holes are several periods away from the corner. The PL spectra are shifted for clarity

To demonstrate the topological protection of the corner state, we fabricated topological cavities without and with defects. Figure 3a shows the PL spectra for defect-free cavities. In this case, fluctuations in both the corner and edge states are observed, which may result from fabrication imperfections. In the current state-of-the-art techniques, fabrication imperfections of approximately 2–5 nm always exist. To estimate the influence of the fabrication imperfections, we calculate the resonance wavelength and Q factor of the cavity with a random perturbation of the size of the air holes of approximately 2–5 nm around the corner (see Supplementary Information). The calculated fluctuation in the resonance wavelength can be up to 6 nm, and the observed fluctuation in the wavelength of approximately 2 nm is within this range. In addition, compared with that of the cavity with no perturbations, the calculated Q factors of cavities with perturbations around corners are decreased by approximately an order of magnitude, which well explains the difference in the Q factor for defect-free cavities between the experiment (10^3^) and theory (10^4^).

Although the Q factor and resonance wavelength of the corner state are susceptible to disorder around the corner, the corner state induced by quantized edge polarizations is topologically protected by the nontrivial 2D Zak phases of the bulk band. Therefore, the corner state exists even with harsh perturbations as long as the topological property of the PhC is not changed. Figure 3b shows the PL spectra of cavities with different amounts of defects in the bulk of the PhC. The defects are created by introducing randomly missing square holes, as shown in the inset. We can see that the corner state still exists even with nine missing holes, with only small fluctuations in the Q factors and wavelengths. The wavelength fluctuation is approximately 2.5 nm, which is comparable to that of the defect-free samples in Fig. 3a. According to the numerical results in the Supplementary Information, the wavelength deviation of cavities with nine missing holes is approximately 0.8 nm, which can be ignored in comparison with the deviation induced by fabrication imperfections. The fluctuation in cavities with defects thus mainly results from fabrication imperfections. Therefore, the existence of a corner state is demonstrated to be robust against defects in the bulk of the PhC.

**Fig. 4:**
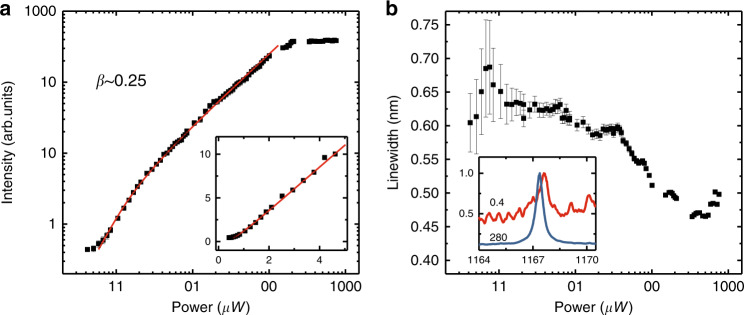
Lasing behaviour of the corner state. **a** Pump-power dependence of the corner state for the cavity with *a*=360nm, *D*=222nm and *g*=30nm, on a logarithmic scale. The inset shows the enlarged curve around the threshold on a linear scale. Squares represent the experimental data, and the line represents the fitted result obtained with the semiconductor laser model. *β* is estimated as approximately 0.25. The lasing threshold is approximately 1µW. **b** Linewidths of the corner state as a function of pump power. The inset shows the normalized PL spectra for different pump powers. The unit of pump power is µW. The linewidth shows a clear narrowing. The linewidths and intensities are both extracted by fitting the high-resolution spectra with Lorentz peak functions

To verify the lasing behaviour of the corner state with QDs as the gain medium, the pump-power dependence of the corner state emission in the topological nanocavities is investigated. We have measured many cavities with different geometric parameters. Only a few cavities show lasing behaviour, while most do not (see Supplementary Information). This may result from the mismatch between QDs and the corner state in the spectral or spatial domain. Figure 4 illustrates the lasing behaviour with high performance from the cavity with *a* = 360 nm, *D* = 222 nm, and *g* = 30 nm. The linewidths and intensities of the corner state are both extracted by fitting the high-resolution spectra with Lorentz peak functions. As shown in Fig. 4a, the light-in-light-out (L–L) plot on the logarithmic scale shows a mild “s” shape, suggesting a lasing oscillation with high *β*. A clear kink is observed in the L–L curve on a linear scale, indicating a low threshold of approximately 1 μW. The β factor, which is approximately 0.25, is extracted by fitting the curve with the semiconductor laser model^50^ (see Materials and Methods). The observed thresholds of our proposed higher-order topological nanolaser are approximately three orders of magnitude lower than those of the current topological edge lasers;^13,15–18^ furthermore, they are nearly 2% of the threshold for the topological nanolaser based on the 0D interface state^19^.

Meanwhile, as shown in Fig. 4b, a clear decline in the linewidth and spectral narrowing are observed with increasing pump power, which further verify the lasing behaviour of the topological nanocavities. It is worth noting that the saturation of intensities and increase of linewidths at high pump power may result from heating in the nanocavities. At a pump power of approximately 0.5 μW, which is below the threshold, the Q factor of the cavity mode is estimated to be approximately 1700. Therefore, the low threshold and high *β* can be attributed to the small volume and high Q factor, which lead to strong optical confinement. Such high performance of the topological nanolaser is comparable to that of conventional nanolasers^24–27^, indicating great prospects in applications with built-in protection.

## Discussion

In conclusion, we demonstrated a topological nanolaser with high performance based on the second-order topological corner state in 2D PhC slabs. The Q factor of the corner state has been optimized by suitably tuning the distance between topologically distinct PhC slabs, which is confirmed both theoretically and experimentally. The existence of the corner state, which is topologically protected by the nontrivial Zak phase of the bulk band, is demonstrated to be robust against defects in the bulk of the PhC. The lasing behaviour with a low threshold of approximately 1 μW and a high *β* of approximately 0.25 is observed at 4.2 K. The operation temperature is lower than that of topological lasers based on multiple quantum wells^13,15^. However, the observed threshold is much lower than that of current topological lasers due to the small mode volume and high Q factor, and the performance is comparable to that of conventional nanolasers. Our result shows an example of downscaling the applications of topological photonics to the nanoscale and demonstrates the great potential of topological nanocavities for applications in topological nanophotonic devices.

## Materials and methods

### Numerical simulation

The Q factor, mode volume and mode profile calculations were calculated by the finite element method in this work. For all the calculations, the actual 3D simulation was employed with 43 × 43 arrays (the same as the experimental sample). Due to the up-down symmetry (we focus on the TE-like modes), we can reduce the memory consumption and increase the running speed by only simulating half of the structure with the help of the applied perfect magnetic conductor boundary condition in the xy-plane. In addition, perfectly matched layer domains are used to decrease the far-field reflection. The refractive index of the GaAs slab is assumed to be *n* = 3.4.

### Fabrication

The samples were grown by molecular beam epitaxy and contained a GaAs slab with a thickness of 160 nm, a 1 μm AlGaAs sacrificial layer and a GaAs substrate. The GaAs slab contained a single layer of InGaAs QDs at the centre with a density of approximately 500 μm^−2^. The topological nanocavities were fabricated by using electron beam lithography followed by inductively coupled plasma and wet etching processes. First, the GaAs slab was spin-coated with a positive resist (AR-P 6200) at 3000 rpm for 60 s. The resist was exposed to a 100 kV electron beam, and the exposure dose was adjusted according to the size of the squares. After developing the exposed resist (AR 600-546), the samples were etched by inductively coupled plasma with gases of BCl3 and Ar. Finally, wet etching with HF solution was used to remove the AlGaAs sacrificial layer to form an air bridge.

### PL measurement

Confocal micro-PL measurements were performed at 4.2 K using a liquid helium flow cryostat. An objective lens with a numerical aperture of 0.7 was used for excitation and collection. The cavities were excited around the corner by a continuous laser with a wavelength of 532 nm. The spot radius was approximately 1–2 μm. In this case, the corner state can be efficiently excited. The PL signals were dispersed by a grating spectrometer and detected with a liquid-nitrogen-cooled charge-coupled device camera with a spectral resolution of 60 μeV.

### Semiconductor laser model

The L–L curves in Fig. 4a, c of the main text were fitted by the conventional semiconductor laser model^19,50^ described below.

$$\frac{{dn}}{{dt}} = - \kappa n + \beta \gamma \left( {N - N_T} \right)n + \beta \gamma N,$$

$$\frac{{dN}}{{dt}} = P - \gamma N - \beta \gamma \left( {N - N_T} \right)n,$$

where *N* and *n* are the numbers of carriers and cavity photons; *P* is the pump power; *κ* is the cavity decay rate; *γ* is the total spontaneous emission rate; *N*_*T*_ is the transparent carrier number; and *β* is the spontaneous emission coupling factor. *κn*, corresponding to the output intensity, is fitted using the experimental data.

### Supplementary information

Supplementary Information

## Data Availability

The authors declare that all data supporting the findings of this study are available within the paper and its Supplementary Information files.
