# Correction to: Low-threshold topological nanolasers based on the second-order corner state

**DOI:** 10.1038/s41377-021-00606-6

**Published:** 2021-08-13

**Authors:** Weixuan Zhang, Xin Xie, Huiming Hao, Jianchen Dang, Shan Xiao, Shushu Shi, Haiqiao Ni, Zhichuan Niu, Can Wang, Kuijuan Jin, Xiangdong Zhang, Xiulai Xu

**Affiliations:** 1 Key Laboratory of advanced optoelectronic quantum architecture and measurements of Ministry of Education, School of Physics, Beijing Institute of Technology, 100081 Beijing, China; 2 Beijing Key Laboratory of Nanophotonics & Ultrafine Optoelectronic Systems, Micro-nano Center, School of Physics, Beijing Institute of Technology, 100081 Beijing, China; 3 Beijing National Laboratory for Condensed Matter Physics, Institute of Physics, Chinese Academy of Sciences, Beijing, 100190 China; 4 CAS Center for Excellence in Topological Quantum Computation and School of Physical Sciences, University of Chinese Academy of Sciences, Beijing, 100049 China; 5 State Key Laboratory of Superlattices and Microstructures, Institute of Semiconductors Chinese Academy of Sciences, Beijing, 100083 China; 6 Songshan Lake Materials Laboratory, Dongguan, Guangdong 523808 China

**Keywords:** Nanocavities, Nanophotonics and plasmonics, Quantum dots

Correction to: *Light: Science & Applications*

10.1038/s41377-020-00352-1 published online 29 June 2020

Following publication of this article^1^, it was noted that Fig. 4a and b contained some errors.

The horizontal coordinates in 4a and b should be corrected to 1, 10, 100, 1000. The correct Fig. 4 is provided in this Correction.

**Figure:**
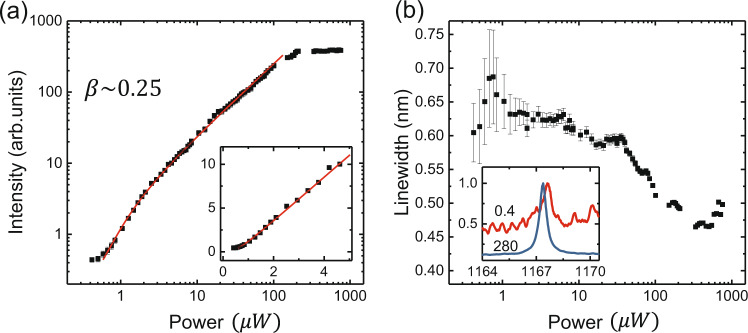
Fig. 4

The original article has been updated.
