# Supplementary material for: Low-threshold topological nanolasers based on the second-order corner state
Source: Light Sci Appl. 2020 Jun 29;9:109. doi: 10.1038/s41377-020-00352-1 (PMC7324580; doi:10.1038/s41377-020-00352-1)
Supplement: Supplementary file 1 — Supplementary Information [file 41377_2020_352_MOESM1_ESM.pdf]

Supplementary Information for Low-threshold topological nanolasers based on the second-order corner state

Weixuan Zhang,[1,2,*] Xin Xie,[3,4,*] Huiming Hao,[5] Jianchen Dang,[3,4] Shan Xiao,[3,4] Shushu Shi,[3,4] Haiqiao Ni,[5] Zhichuan Niu,[5,†] Can Wang,[3,4,6] Kuijuan Jin,[3,4,6] Xiangdong Zhang,[1,2,‡] and Xiulai Xu[3,4,6,§]

[1]*Key Laboratory of advanced optoelectronic quantum architecture and measurements of Ministry of Education, School of Physics, Beijing Institute of Technology, 100081, Beijing, China*

[2]*Beijing Key Laboratory of Nanophotonics & Ultrafine Optoelectronic Systems, Micro-nano Center, School of Physics, Beijing Institute of Technology, 100081, Beijing, China*

[3]*Beijing National Laboratory for Condensed Matter Physics, Institute of Physics, Chinese Academy of Sciences, Beijing 100190, China*

[4]*CAS Center for Excellence in Topological Quantum Computation and School of Physical Sciences, University of Chinese Academy of Sciences, Beijing 100049, China*

[5]*State Key Laboratory of Superlattices and Microstructures, Institute of Semiconductors Chinese Academy of Sciences, Beijing 100083, China*

[6]*Songshan Lake Materials Laboratory, Dongguan, Guangdong 523808, China*

(Dated: June 10, 2020)

I. THEORY MODEL AND CALCULATION

SSH model is a typical example to construct one dimensional chiral Hamiltonian with nontrivial topology, which is characterized by the winding number. It is a chain consisting of two sublattices with alternating coupling strengths, which can be distinguished as inter-cell (t') and intra-cell coupling strengths (t)[1], as shown in Fig. S1(a). When $t < t'$ ($t > t'$), a nontrivial (trivial) winding number can be achieved, and therefore a topological edge state will exist at the interface between chains with different winding numbers[2]. The SSH model can be extended to the 2D case, whose topology is described by the 2D Zak phase $\theta^{Zak} = (\theta_x, \theta_y)$. It is defined as

$$\theta_i = \int \mathrm{d}k_x \mathrm{d}k_y Tr[A_i(k_x, k_y)], \tag{1}$$

where $i = x$ or y and $A_i(k_x, k_y)$ is the Berry connection[3]. Here, inspired by the 2D SSH model to construct the 0D corner state, we designed two kinds of photonic crystal (PhC) slabs, which possess the identical band structure but different 2D Zak phases, as shown in Fig. S1(b). The structure on the left (right) side of Fig. S1(b) possesses the trivial (nontrivial) 2D Zak phase of (0,0) ((π, π)). The band structure of the two PhC is shown in the inset of Fig. S2(a). We can see that a bandgap appears in the range of [250 THz, 287 THz] (brown area). According to the bulk-edge correspondence, the quantized bulk dipole polarization which is characterized by the nontrivial 2D Zak phase will protect the existence of topological edge state with the nontrivial edge dipole polarization. In the same way, the topology of edge state is related to the quantized edge dipole polarization, which will protect the existence of corner state. That is, the corner state exists as long as $\theta_x^{Zak} = 0.5$ and $\theta_y^{Zak} = 0.5$. Thereby, a second-order topological corner state can be formed at the intersection between two topologically distinct structures, as shown in Fig. 1(a).

The PhC structures used here have the long-range and complex interactions, which are different from the SSH model with only the nearest-neighbour coupling. The 2D SSH mode preserves the chiral symmetry and yields a symmetric spectrum with respect to the zero-energy. There is no bandgap, and the corner mode is fixed at the zero-energy and embedded within the symmetric bulk states. Due to the degeneracy with bulk states, the corner state can be severely affected by disorder and perturbations. In contrast, in our PhC structures, the long-range and complex interactions break the chiral symmetry and modify the band structure, making the corner state move out of the bulk band and fall inside the bandgap, as

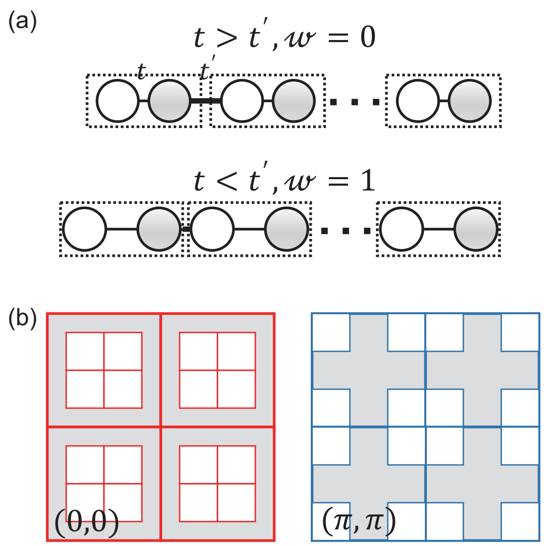

Fig. S 1. SSH model. (a) Schematic of SSH model. It consists of two sublattices indicated by different colors, having alternating coupling strength t and t'. For chain above (below), $t > t'$ ($t < t'$) and the winding number is 0 (1). (b) Generalized 2D SSH model in the main text. The structures on the left and right correspond to the 2D Zak phase of (0,0) and (π, π), respectively.

shown in Fig. S2(a)-(c). Therefore, the corner mode in topological PhC cavities, protected by the nontrivial topological bandgap, is robust against the bulk disorders. Fig. S2(d)-(f) present the corresponding electric field profiles of the corner mode, one of edge modes and one of bulk modes with $g = 10$ nm. With increasing g, the corner state and edge states both show red-shift. However, the corner state locates within the gap while the edge states gradually get merged within the bulk modes. Additionally, the corner state can be well isolated from other modes in spectrum, so it is possible to investigate the properties of the corner state by fluorescence spectroscopy.

Although the existence of corner state is protected by the topology of bulk of PhC, the Q factor and resonance wavelength are susceptible to the perturbations around corner. In order to estimate the influence of fabrication imperfections on the corner state, we calculated the Q factor and resonance wavelength of corner states in two cavities with different perturbations around the corner, as shown in Fig. S3(a). The perturbations are introduced by randomly changing the length of square holes (about 2-5 nm) around the corner, which is the same as the deviation induced by the fabrication imperfection with the state-of-the-art techniques.

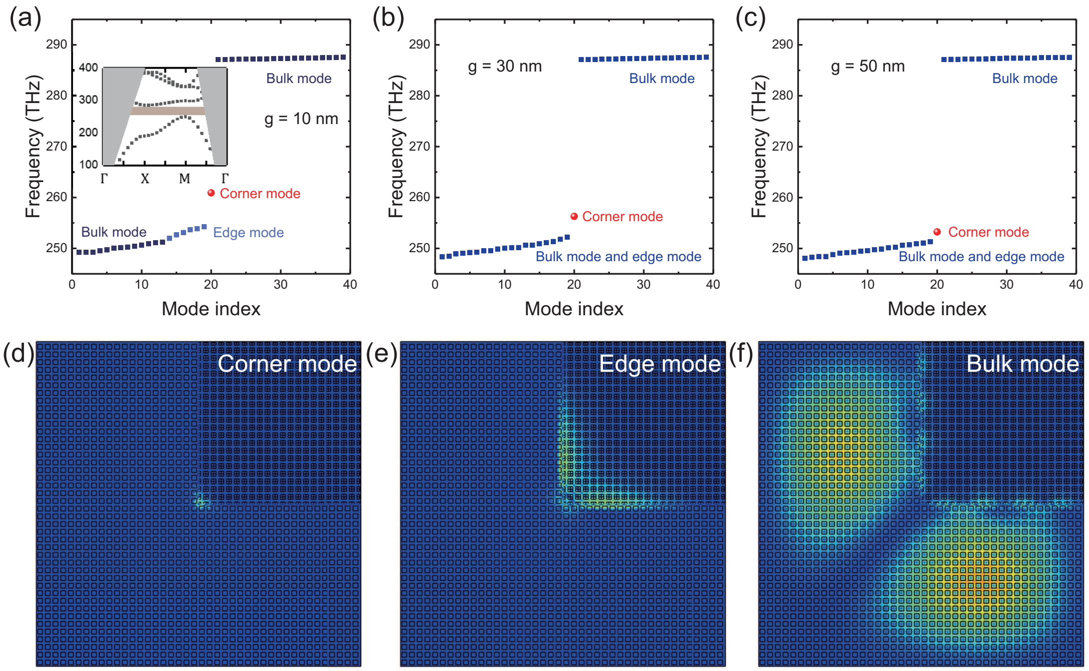

Fig. S 2. Calculation results of different modes. The eigen-spectrum of the topological photonic nanocavity with (a) $g = 10$ nm, (b) $g = 30$ nm and (c) $g = 50$ nm. The inset in (a) shows the band structure of PhC. Electric field profiles of (d) corner mode, (e) one of edge modes and (f) one of bulk modes for cavity with $g = 10$ nm.

The resonance wavelength shows a deviation up to 6 nm and the Q factor can be decreased by an order of magnitude in comparison to perturbation-free cavity, whose Q factor is about 50,000. Different from the case with the perturbation around the corner, there is nearly no deviation on the Q factor and resonance wavelength by introducing nine missed square holes in the bulk of PhC, as shown in Fig. S3(b). This phenomenon indicates the robustness of corner state with respect to defects in bulk of PhC.

II. SEMICONDUCTOR LASER MODEL

Fig. S4(a) shows the L-L curves with various values of β calculated by a conventional semiconductor laser model[5,6]. For small β, the L-L curves show a clear kink. With increasing β, the kink gradually becomes smoother. Fig. S4(b) shows the calculated L-L curves with different values of N_T. It is clearly shown that the threshold becomes larger with increasing N_T.

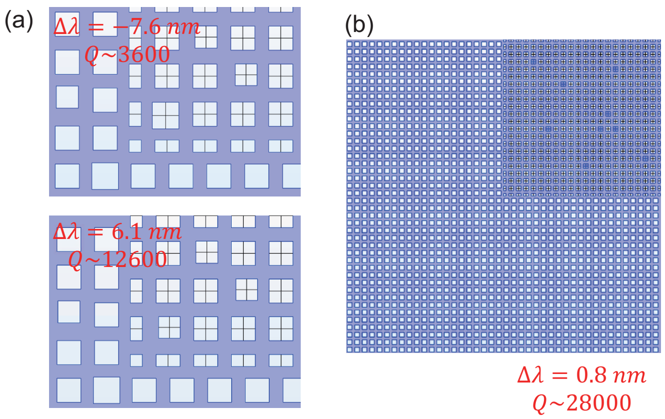

Fig. S 3. Calculation results of cavities with perturbations and defects. (a) Calculated results of corner state in two cavities with different perturbations around corner. The perturbations in two cavities are both introduced by random deviation of 2-5 nm. The resonance wavelength and Q factor for perturbation-free cavity are 1183.8 nm and 50,000, respectively. (b) Calculated results of corner state in cavity with nine missing square holes. The results are calculated by the finite element method[4].

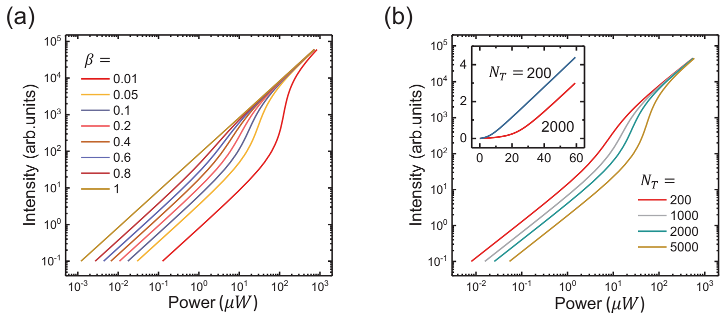

Fig. S 4. Calculated L-L curves. (a) L-L curves for various values of β. (b) L-L curves with different values of N_T. Inset shows the curve with two values of N_T on a linear scale.

III. NONLASING BEHAVIORS

Fig. S5 shows two examples of non-lasing behaviours in the topological nanocavities. As we can see from Fig. S5, the L-L curves exhibit no kinks and the linewidths don't show apparent narrowing and begin to increase even at small pump power, which have great difference from the lasing behaviours in the main text. In our work, we have measured thousands of cavities and only a few cavities show lasing action. Most of the fabricated

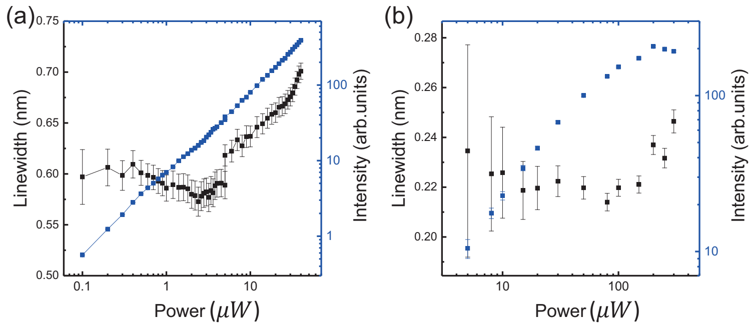

Fig. S 5. Non-lasing behaviours for two topological cavities. Blue and black points represent the measured intensity and linewidth, respectively.

cavities didn't lase. This may mainly result from the mismatch between QDs and corner state in the spatial or spectral region. On the one hand, the hundreds of cavities have different parameters including periods, length of square hole and g. It causes that the corner states of these cavities have a wide range of distribution in energy. On the other hand, the QDs are randomly distributed in energy and position, and most of them are etched during the fabrication process. Therefore, in most of these cavities, it is difficult to efficiently couple QDs with corner state due to the spectral or spatial mismatch. Because of this reason, some cavities, even with a high Q about 5000, didn't show lasing action. Meanwhile, we found that the lasing action is apt to appear in the corner state with the wavelength of about 1165-1185 nm, where the density of QDs is much larger. In that case, it is easy to get a better spectral match with QDs. Therefore, in our work, whether the lasing action appears or not is not only related to the Q, but also associated with the resonant wavelength of the corner state.

---

[*] Contributed equally to this work.

[†] zcniu@semi.ac.cn

[‡] zhangxd@bit.edu.cn

[§] xlxu@iphy.ac.cn

[1] Su, W. P., Schrieffer, J. R. & Heeger, A. J. Solitons in polyacetylene, Physical Review Letters **42**, 1698 (1979).

[2] Ryu, S. & Hatsugai, Y. Topological origin of zero-energy edge states in particle-hole symmetric systems, Physical Review Letters **89**, 077002 (2002).

[3] Zak, J. Berry's phase for energy bands in solids, Physical Review Letters **62**, 2747 (1989).

[4] Strang, G. & Fix, G. J. *An analysis of the finite element method*, Vol. 212 (Prentice-hall Englewood Cliffs, NJ, 1973).

[5] Bjork, G. & Yamamoto, Y. Analysis of semiconductor microcavity lasers using rate equations, IEEE Journal of Quantum Electronics **27**, 2386–2396 (1991).

[6] Ota, Y. *et al.* Topological photonic crystal nanocavity laser, Communications Physics **1**, 86 (2018).
